# Novel Role Of DSNB in Staging of Primary Urethral Cancer: New Standard?

**DOI:** 10.1590/S1677-5538.IBJU.2025.9905

**Published:** 2025-01-31

**Authors:** Adnan Fazili, R. Barry Sirard, Laura Elst, Kaat Vandermaesen, Hongzhi Xu, Maarten Albersen, Philippe E. Spiess

**Affiliations:** 1 H. Lee Moffitt Cancer Center Department of Genitourinary Oncology Tampa FL USA Department of Genitourinary Oncology, H. Lee Moffitt Cancer Center, Tampa, FL, USA;; 2 University of South Florida Morsani College of Medicine Tampa FL USA University of South Florida Morsani College of Medicine, Tampa, FL, USA;; 3 University Hospitals Leuven Department of Urology Leuven Belguim Department of Urology, University Hospitals Leuven, Leuven, Belgium;; 4 Department of development and regeneration Leuven Belguim Department of development and regeneration, KU Leuven, Leuven, Belgium

**Keywords:** Urethral Neoplasms, Sentinel Lymph Node Biopsy

## Abstract

We describe the novel use of dynamic sentinel node biopsy (DSNB) in five patients with primary urethral squamous cell carcinoma (U-SCC) and no evidence of inguinal node disease across two centers in North America and Europe between 03/2021 and 06/2024. Each of these referral centers sees over 75 cases of penile cancer per year and approximately 10 cases of U-SCC per year. Patients underwent DSNB concomitant to surgical resection of the primary tumor (n=3), or in a deferred manner (n=2), six weeks after primary surgery. In the five DSNBs performed, clinically occult nodal metastasis was discovered in one patient. In this patient DSNB was performed after local recurrence and repeat imaging confirming cN0 status. Only one minor complication with DSNB was observed. Awaiting further investigations in larger series, this study highlights the feasibility of DSNB in primary U-SCC with clinically node negative disease.

## INTRODUCTION

### Case series

Urethral squamous cell carcinoma (U-SCC) is a rare disease (1). Despite similarities in histology and lymphatic drainage with penile squamous cell carcinoma (PSCC), the clinical recommendations in management of inguinal lymph nodes are different and less defined. Current EAU and NCCN guidelines do not support diagnostic sentinel lymph node surgical excision or prophylactic superficial inguinal lymph node dissection (ILND) for primary urethral carcinoma in the setting of patients that are cN0, regardless of primary stage (2, 3). Werntz et al., studied the node positive rates in 86 cN0 patients with U-SCC undergoing prophylactic inguinal lymph node dissection (ILND), demonstrating only a 9% lymph node positive rate in pT1-T4, cN0 tumors, although rate of pN+ was not risk stratified by primary T stage (4).

However, it is well-established that nodal metastasis is one of the strongest predictors of recurrence and survival in primary urethral carcinoma (5-7). By the time patients have clinically evident nodes, treatment strategies are often multidisciplinary, with limited curative potential. In this context, the value of early identification of patients at risk of harboring early nodal metastasis cannot be overstated. Herein, we share a case series utilizing a previously unreported dynamic sentinel node biopsy (DSNB) technique in patients with clinically node negative primary U-SCC.

We identified five patients with cN0 U-SCC (all were confirmed to be primary urethral tumors by genitourinary pathologists). Tumoral characteristics and DSNB protocol are shown in Table-1. 99mTc-nanocolloid was injected subcutaneously, immediately proximal to tumor. A dynamic recording was started immediately following tracer injection with the detector positioned anteriorly at the level of the pelvis. This procedure was repeated until a SN was visible (uni- or bilaterally) up to a maximum of 60 minutes. Finally, a SPECT/CT of the pelvis was performed to detect and localize the SNs. Late imaging (static planar and/or SPECT/CT) was acquired, with or without additional tracer injection, if no SN was visible after the initial imaging phase. Initial radiotracer dosing was not standardized. Intraoperatively, subcutaneous isosulfan blue dye was injected in 3 cases and free indocyanine green (ICG) was used in 2 cases. The number and location of sentinel nodes were described as first echelon if within Dressler's Triangles (primary echelon), above the inguinal ligament (secondary echelon) and pelvic nodes (tertiary echelon). SNs were pursued using a gamma probe (Neoprobe, Johnson & Johnson Medical) and fluorescence camera (FIS-00, Photodynamic eye, Hamamatsu Photonics) in cases utilizing ICG. All harvested nodes were embedded in sections of 2mm thickness and screened with H&E staining. If the SN seemed negative for carcinoma during initial screening, deeper sections of 500 microns were made and stained for prekeratin (AE1/AE3). Follow up consisted of physical examination and inguinal imaging every 3 months during the first 2 years after DSNB.

**Table 1:** Outcomes for patients undergoing DSNB for cN0 primary urethral carcinoma. Pt = Patient.

	P1	P2	P3	P4	P5
Age at Diagnosis (yrs)	71	57	67	85	67
History of urethral stricture	No	No	Yes	No	No
History of CIC	No	No	Yes	No	No
Smoking status	Former	Never	Current	Never	Never
Primary tumor location	Distal urethra	Bulbar urethra	Bulbar urethra	Bulbar urethra	Distal urethra
Primary tumor investigation	Cysto	Cysto+MRI	Cysto+MRI	Cysto	Cysto
HPV status	Positive	Negative	Negative	Negative	Positive
Primary surgery	PP+DU	TP+TU	TP+TU	PP+DU	PP+DU
pT stage	2	3	3	2	3
Histology	NA	NA	NA	NA	Warty-Basaloid
Grade	3	2	2	3	2
Margin status	Invasive	Negative	Negative	Negative	Negative
Inguinal node investigation	US+PET/CT	US+PET/CT	US+ CT	CT	MRI
DSNB radiotracer	99mTc-COL	99mTc-COL	99mTc-COL	99mTc-COL	99mTc-COL
Dosage (MBq)	80	120	60	16.9	16
Injection site	Adjacent	Adjacent	Adjacent	Adjacent	Adjacent
Adjunct dye	Isosulfan blue dye	Isosulfan blue dye	Isosulfan blue dye	Free ICG	Free ICG
Primary Imaging of SN	NM SPECT/CT	NM SPECT/CT	NM SPECT/CT	NM SPECT/CT	NM SPECT/CT
# of SN on imaging (R)	3	1	1	1	4
# of SN on imaging (L)	0	1	1	1	2
Location of SN (R)	10, 20	10	10	10	10
Location of SN (L)	Non-visible	10	10	10	10, 20
# nodes harvested (R)	3	1	1	1	6
# nodes harvested (L)	0	2	1	1	1
DSNB pN stage	0	0	0	0	III
Complication	None	None	None	None	Lymphocele

CIC = Clean intermittent catheterization. MRI = Magnetic resonance imaging. US = Ultra-sonography. CT = Computed tomography PET = Positron emission tomography. 99mTc-nanoCOL = Technetium nanocolloid nuclear isomer.

NM SPECT = Nuclear Medicine single-photon emission computed tomography. PP = Partial Penectomy. TP = total penectomy. DU = Distal urethrectomy TU = Total penectomy.

**Figure 1:**
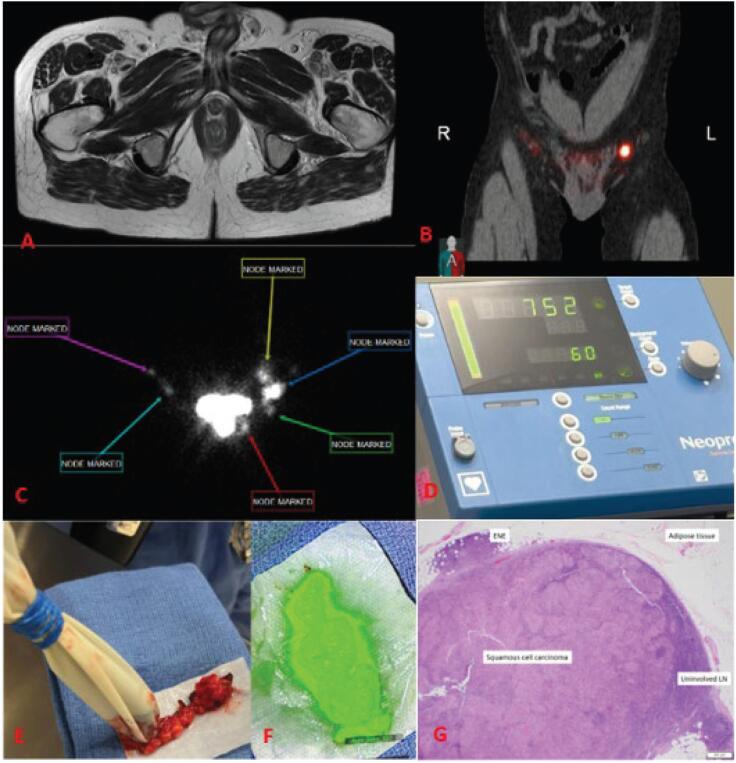
DSNB protocol for primary urethral squamous cell carcinoma.

One patient was found to have a DSNB positive for disease 7 months after initial diagnosis and 1 month after local recurrence. His initial treatment was a partial penectomy and distal urethrectomy revealing a pT3a, Grade 2, primary urethral carcinoma with negative surgical margins. At the time of LR, repeat contrasted MRI revealed no regional lymphadenopathy (cN0). LR was treated with total penectomy and urethrectomy. DSNB patient revealed 1/7 nodes positive with a unifocal 6mm with extranodal extension (Figure-1). He underwent a bilateral superficial and deep inguinal lymphadenectomy with all 8 nodes negative. He remains clinically disease free since DSNB without receipt of adjuvant therapy.

A) Axial T2 MRI of patient prior to planned DSNB showing no regional lymphadenopathy (cN0);

B-C) Tomographic and planar SPECT/CT images obtained after administration of 425 microcuries of Technetium-99m sulfur-colloid via subcutaneous injection into the cancer site involving the penis. Bilateral highly avid sentinel nodes and less avid secondary echelon nodes demonstrated.;

D-F) Example of ex vivo analysis of removed nodes: Intraoperative use of hand probe continually assessing nuclear activity. Indocyanine green (ICG) dye illumination under infrared source to evaluate suspected lymph nodes.

G) Pathologic analysis of DSNB specimens revealing positive left inguinal node with extra-nodal extension (ENE).

## DISCUSSION

Urethral squamous cell carcinoma (U-SCC) appears to be a separate entity from PSCC despite similarities in histology, location and lymphatic drainage. Unlike PSCC, where prophylactic ILND for cN0 disease has shown survival benefit, early ILND has no current role in cN0 U-SCC (8). There are very few studied examining inguinal nodal management of male U-SCC. The largest study examining nodal management of primary U-SCC in men found that ILND was only associated with an improvement in overall survival in cN+ patients (4). This study did not differentiate pN+ rates in cN0 patients based on primary T stage and grade, which questions whether a certain subset of cN0 patients with primary urethral cancer may benefit from invasive regional node sampling.

The prognosis of node positive, pure squamous cell urethral carcinoma remains unknown and studies assessing the impact of histology on outcomes in primary urethral carcinoma are limited. Studies including patients with urothelial, squamous cell and adenocarcinoma histology report overall survival outcomes of primary urethral carcinoma of all histologic types at 5 and 10 years at 46% and 29%, respectively, and report that the presence of nodal spread independently predicts an increased likelihood of death [HR 2.07 (1.17-3.67) p=0.013] (5). A study of 46 males with predominantly squamous cell carcinoma of the urethra by Dalbagni et al. identified squamous cell and epidermoid histology as a predictor of worse overall and cancer specific survival (p=0.0001) and reported 5 years overall and disease specific survival rates at 42% and 50% respectively (6). In this study we demonstrate that DSNB can identify patients with early nodal spread and provide an opportunity for early curative therapy.

We highlight the similarities and differences of DSNB technique between the two institutions. The choice of radiotracer and imaging nodality to detect sentinel nodes was identical. Interestingly, the European technique incorporates the use of inguinal ultrasound to further select patients for DSNB. Only patients without suspect lymph node on ultrasound or patients with questionable nodes on ultrasound but negative fine needle aspiration cytology (FNAC) are selected for DSNB. This additional measure is interesting and studies from the Netherlands Cancer Institute have been shown this to potentially enhance the sensitivity of DSNB (9). At our institution, provided patients had no suspicious inguinal nodes on axial imaging obtained within 6 weeks of the procedure, we prefer to use free unbound indocyanine green injection peri-urethrally and evaluate nodes intraoperatively using real time infrared photography. Only nodes that are intraoperatively radioactive and ICG positive are resected. In contrast, our colleagues in Europe use methylene blue, allowing visualization of blue dye within lymphatic channels and sentinel nodes. Collaborative efforts to standardize techniques will be beneficial in providing a uniform guideline for successful DSNB.

We hypothesize that DSNB alone may potentially cure a subset of patients with micro-metastatic disease, while discovering and selecting patients with more aggressive disease for early curative measures. Penile cancer and urethral cancer are thought to exhibit predictable stepwise lymphatic metastasis from inguinal to pelvic and para-aortic nodes. Distant metastasis is atypical without concurrent LN spread (10, 11) Radical LN surgery remains the cornerstone of management for early nodal disease (cN1–2) but is associated with significant morbidity and the benefit of adjuvant treatments is uncertain. Nodal surgery alone is often not curative in cases of extensive LN involvement. Therefore, multimodal treatment approaches with (neo)adjuvant chemotherapy and/or radiotherapy (RT) are often considered (12-14). In current guidelines, neoadjuvant chemotherapy is recommended in patients presenting bulky inguinal disease, pelvic metastases on imaging. In the group of patients without radiological signs of pelvic LN involvement with two or more tumor-positive ipsilateral inguinal LN metastasis or the presence of extranodal extension (ENE), surgical treatment of the pelvis (so-called prophylactic pelvic treatment) is recommended.

Clearly, the paradigm of clinical care for primary urethral cancer continues to evolve and we suspect the application of DSNB may represent a further refinement in clinical care. We encourage prospective validation of our work as an international collaborative effort, possibly through organizations like the Global Society of Rare Genitourinary Tumors. We hope that the novel use of this technique is further investigated as a diagnostic and treatment standard in those with locally advanced (pT2-4) urethral cancer in the setting of cN0. DSNB, with its relatively low complication rates, may represent an accommodating middle ground between the need for diligent surveillance with physical exam and imaging in an effort to avoid occult nodal metastatic progression versus potentially morbid ILND surgery which patients and physicians alike are hesitant to propose without a clear benefit it can offer.

## Data Availability

Data is available upon request from the corresponding author.
